# Rationale and methods of the ‘Northern Ireland Youth Wellbeing Survey’ and initial findings from the Strengths and Difficulties Questionnaire

**DOI:** 10.1177/13591045221075525

**Published:** 2022-03-01

**Authors:** Lisa Bunting, Claire McCartan, Gavin Davidson, Anne Grant, Ciaran Mulholland, Dirk Schubotz, Orla McBride, Jamie Murphy, Mark Shevlin

**Affiliations:** 1 1596 Queen’s University Belfast, Northern Ireland; 2 2596 Ulster University, Northern Ireland

**Keywords:** Mental health, children and young people, prevalence, Northern Ireland, Northern Ireland youth wellbeing survey

## Abstract

*Backgrounds and Aims:* The Northern Ireland Youth Wellbeing Survey (NIYWS) was commissioned by the Health and Social Care Board (NI) with the aim of providing reliable prevalence estimates of the mental health problems of children and young people aged 2–19 years. *Method:* The NIYWS used a random probability design, stratified by deprivation decile and county, to ensure even geographical distribution and representation. The survey used a broad range of validated measures to identify children and young people who met established clinical criteria for common mood, anxiety and behaviour disorders, trauma related disorders, as well as those at risk of autism spectrum disorder, eating disorders, future psychotic illness, self-injury or suicide. *Results:* Data were collected on 3074 children and young people aged 2–19 years, as well as over 2800 parents. The survey achieved a high response rate (67%) and initial findings indicated that 11% of the sample were at risk of emotional or behavioural problems. *Conclusions:* The NIYWS was the first large scale nationally representative survey of the mental health of children and young people in NI. Despite the legacy of political violence the initial findings show comparable levels of emotional and behavioural problems to England.

Mental health problems contribute significantly to the global disease burden and are major causes of disability, suicide and physical health problems (WHO, 2017). The prevalence of mental health problems has risen steadily for over 50 years (Collishaw et al., 2004), with between 50–75% of adult mental disorders beginning before the age of 18 years (Kessler et al., 2007; McGorry & Mei, 2018). In the UK, the Mental Health of Children and Young People Survey (MHCYPS) has been providing prevalence rates of mental health problems for young people since 1999 (Ford et al., 2003), with subsequent surveys in 2004 (McGinnity et al., 2005) and 2017 (Sadler et al., 2018). The MHCHYP (2017) England survey found that one in eight (12.8%) 5–19 year olds had at least one clinically diagnosable mental health disorder, with one in 12 (8.1%) having an emotional disorder such as anxiety or depression and one in 20 (4.6%) having a behavioural or ‘conduct’ disorder.

Whilst prevention and early intervention approaches in physical health are well established, similar approaches in mental health have been less well developed (McGorry & Mei, 2018) despite evidence of their effectiveness (Correll, et al., 2018). Although young people and their families should expect that appropriate services are available when they develop distressing mental health problems, young people have the worst levels of access to mental health care across the lifespan (McGorry & Mei, 2018). The planning and commissioning of health and social care services should be based on the best available evidence and an accurate assessment of the scale and nature of mental health problems in a population must be the keystone for developing such services and responding to need.

This primary aim of this paper is to describe the methodology of the first ever epidemiological study in Northern Ireland to assess the prevalence of child and adolescent mental health problems at a national level – the Northern Ireland Youth Wellbeing Survey (NIYWS). In addition, its presents some initial headline results indicating the levels of emotional and behavioural problems, based on the Strengths and Difficulties Questionnaire (Goodman, 1997), stratified by age and gender. These are considered in the context of recent UK research from HCYP (2017) England.

## Methods

### Study design and aims

The NIYWS is a stratified random probability household survey, funded the Department of Health NI, commissioned by the Health and Social Care Board (HSCB) NI, and undertaken by a consortium comprised of researchers from Queen’s University Belfast, Ulster University and the Mental Health Foundation. The study aims were:

1. To collect reliable and valid data on the prevalence of mental health disorders among 2–19 year olds in Northern Ireland;
2. To provide estimates of prevalence of common mental health disorders, and estimate levels of other psychological problems, for children and young people in Northern Ireland.
3. To estimate the association between demographic, social, familial, and stress-related risk factors and mental health disorders and psychological problems.
4. Use the results to help inform mental health policy, planning and service development.

### Sampling

Children and young people were eligible to take part if they were aged 2–19 and lived in Northern Ireland. To produce reliable estimates of mental disorder prevalence, based on a population prevalence of 7.5% (the lifetime prevalence of psychotic like experiences: McGrath et al., 2015) with a confidence level of 95%, and precision of 1%, a target sample size of 2596 children and young people was identified. Due to caution about the possible implications of the UK General Data Protection Regulation (2018) the research team were not given permission to access the NHS Patient Register, Child Benefit Register or other data registers that could have reliably indicated households with children eligible to participate in the study. As a result, it was necessary to randomly select addresses from households across Northern Ireland using the Pointer Database (a postcode register of all households in Northern Ireland) and, at the fieldwork stage, identify a household as either eligible or ineligible, following a visit from one of the interviewing team. Based on a conservative estimate that one in five households in NI had a resident child aged 2–19 years (NI Statistics and Research Agency, 2012), and assuming a response rate of 50%, an initial sample of 30,000 was identified as necessary to achieve a final sample of 3000 completed interviews.

Pointer Database provided information on a total of 989,639 addresses. Addresses were excluded if they were recorded as

• Non-domestic/non-postal (i.e. does not receive post)/other/blank
• Demolished/derelict/under construction/none
• Having a trading or business name of an organisation within an addressable property
• Containing the word ‘FOLD’ in the sub-building name, building name or primary thoroughfare.

The remaining 762,264 eligible addresses where then linked to Northern Ireland’s 2017 Multiple Deprivation Measures data (NI Statistics and Research Agency, 2017) and stratified by deprivation decile and county to ensure even geographical distribution and representation of both affluent and less affluent neighbourhoods. A total of 30,000 addresses were then randomly selected; 25,000 for the main sample and 5000 for the reserve sample. Addresses were issued in six instalments (*n* = 5000) from June 2019 to February 2020, and then clustered according to Electoral Ward to allow for a more efficient fieldwork process.

Due to a higher than expected response rate, it was not necessary to issue the reserve sample.

### Questionnaire development

The survey questionnaire aimed to provide prevalence estimates for the proportion of children and young people at risk of emotional and behaviour problems, those who met established clinical criteria for common mood, anxiety and behaviour disorders, as well as trauma related disorders such as post-traumatic stress disorder and complex posttraumatic stress disorder. It also included a number of screening measures aimed at identifying the proportion of children and young people at risk of autism spectrum disorder, eating disorders or future psychotic illness, as well as measures of self-injury and suicidal thoughts or attempts. An assessment of current parent mental health functioning was also included. The measures used to ascertain the prevalence of mental health problems, mental health exposure and exposure to trauma and adversity are described in detail in the following section. Additionally, a range of behaviours and experiences associated with child mental health and wellbeing, such as social media use, bullying/cyberbullying and alcohol/drug/tobacco use, were also included in the questionnaire.

The selection of potential study measures to include in the survey required finding a difficult balance between: the need to cover the range of mental health problems; the importance of including wider social and contextual issues; the identified research need for more in-depth data on certain issues; what participants might be willing to tolerate; and the available resources. When developing the questionnaire, cognisance was taken of the age of the child/young person and the appropriateness of the questions that could be asked. This resulted in the development of a number of versions of the questionnaire, with the type of interview dependent on the age of the sampled child or young person (Table 1). Parents of children aged 2–10 years completed a short parent survey providing information on child and family demographics, parent mental health, experience of adverse childhood experiences and the ‘Troubles’, followed a questionnaire which measured the mental health and wellbeing of their child. Parents of 11–15 years completed the parent survey whilst the child/young person completed the child mental health questionnaire themselves. For young people aged 16–19 living at home, parents were also asked to complete the parent survey and young people completed the child mental health questionnaire themselves. Where a 16–19 year old living in the parental home did not want their parent or guardian to participate, or the parent or guardian refused to participate, the young person was asked additional demographic questions. Similarly, 16–19 year olds living independently were asked additional demographic questions.

**Table 1.** Type of interview for children and young people of different ages.

Group	Parent	Child
Group A 2–10 year olds	Parent/Guardian interview only (interviewer administered and self-completion)	NA
Group B 11–15 year olds	Parent/Guardian interview (interviewer administered and self-completion)	Child/Young person interview (interviewer administered and self-complete)
Group C1 16–19 year olds	Parent/Guardian interview (interviewer administered and self-completion)	Child/Young person interview (interviewer administered and self-complete)
Group C2 16–19 year olds, parental refusal	Shortened version of parent/Guardian interview answered by young person (interviewer administered and self-completion)	Child/Young person interview (interviewer administered and self-complete)
Group D 16–19 year olds, living independently	Shortened version of parent/Guardian interview answered by young person (interviewer administered and self-completion)	Child/Young person interview only (interviewer administered and self-complete

An initial pilot of the survey questionnaire was undertaken in May 2019 with a small group of respondents (*n* = 20) covering each of the survey groups used for the study. Following feedback from this initial questionnaire testing, a full pilot took place in June 2019 (*n* = 200). This second pilot enabled testing of the flow, content and timings of the complete interview process, together with the operation of fieldwork procedures. As it only highlighted the need for very minor amendments to the questionnaire, the pilot sample was included as part of the final sample. The language used in some of the standardised measures did raise some concerns in the research team and as part of the pilot. It was agreed that the measures should not be altered in case that might have an impact on their psychometric properties but that the contested nature of some of the language would be acknowledged when reporting the findings.

### Mental health problems and disorder measures

#### Child and young people mental health measures

Emotional and behavioural problems: The Strengths and Difficulties Questionnaire (Goodman, 1997) was used to identify children and young people at risk of emotional and behavioural problems. The SDQ is a short 25-item screening questionnaire for use with children, young people and parents. It provides a total difficulties score, as well as measuring five distinct dimensions: conduct problems; emotional symptoms; hyperactivity; peer problems and prosocial behaviour. Each item is scored on a scale of 0 ‘Not true’, 1 ‘Somewhat true’ and 2 ‘Certainly true’ producing subscale scores with a range of 0–10 and these scores where then categorised as ‘Low’, ‘Slightly raised’ and ‘High’ according to the cut-scores proposed by Goodman (1997). The reliability and validity of the SDQ scores have assessed extensively (see Kersten, et al., 2016).

Mood and anxiety disorders: The Revised Children’s Anxiety and Depression Scale (RCADS; Chorpita et al., 2000) is a 47-item questionnaire, that can be self-completed or parent completed, and produces indications of clinically relevant levels of severity of six disorders derived from the diagnostic criteria of the DSM-IV: Major depressive disorder (MDD), separation anxiety disorder (SAD), social phobia (SP), generalised anxiety disorder (GAD), panic disorder (PD) and obsessive compulsive disorder (OCD). The RCADS items are scored on a scale corresponding to 0 ‘Never’, 1 ‘Sometimes’, 2 ‘Often’ and 3 ‘Always’. The RCADS converts raw scores to T-scores, and applies cut-off scores to identify potential ‘clinical thresholds’ (Chorpita et al., 2005).

Conduct and oppositional defiant disorders: The ‘Opposition/Defiant’ subscale of the Autism-Tics, ADHD and other Comorbidities questionnaire (A-TAC; Hansson et al., 2005) was used to provide information on levels of Oppositional Defiant Disorder and Conduct Disorder. The subscale consists of 10 questions, five of which relate to Oppositional Defiant Disorder (ODD) and five which relate to Conduct Disorder (CD) and response options are 1 ‘Yes’, 0.5 ‘Yes, to some Extent’ and 0 ‘No’. The ODD and CD scales have been shown to have good to acceptable internal consistency (Hansson et al., 2005), with a cut-off of ≥3 yielding a sensitivity of 0.51 and a specificity of 0.96 for ODD and a cut-off of ≥2 yielding a sensitivity of 0.55 and a specificity of 0.98 for CD (Kerekes et al., 2014).

Stress-related disorders, trauma and adversity: The Child and Adolescent version of International Trauma Questionnaire (ITQ-CA: Cloitre, et al., 2018) was used to assess posttraumatic stress disorder (PTSD) and Complex PTSD CPTSD among 11–19 years olds. The ITQ-CA consists of six items which measure the symptoms of PTSD and six items which measure the symptoms of CPTSD, together with two items which each assess functional impairment. Participants were asked to indicate the extent each symptom bothered them during the past month using a 5-point Likert scale from 0 (‘Never’) to 4 (‘Almost always’). The items was scored as clinically significant if it was ≥2 and included the presence of at least one symptom from each PTSD cluster and at least one indicator of functional impairment. CPTSD requires the diagnostic criteria for PTSD to be met and in addition at least one symptom from each CPTSD cluster and at least one indicator of functional impairment is required. The internal reliability of the ITQ-CA has been acceptable based on a large adolescent sample: total scale (α = .87), PTSD symptoms (α = .79), and DSO symptoms (α = .86: Kazlauskas et al., 2020).

The ITQ-CA was used in conjunction with the traumatic events checklist, the Child and Adolescent Trauma Screen (CATS; Sachser et al., 2017), which is used to assess the young person’s exposure to traumatic events which may have led to PTSD or CPTSD. The CATS was also adapted to include additional questions on experiences of family trauma and maltreatment, as well as other common family difficulties such as parent separation, parent substance abuse, domestic violence, parent mental health problems and parental incarceration. Generally referred to as ‘adverse childhood experiences’ (ACEs), these are consistently identified as significantly increasing the likelihood of a broad range of negative outcomes amongst adults, as well as children and adolescents (Oral et al., 2016).

The NIYWS measured the 10 ACE categories set out in the original ACE study (Felitti et al., 1998). The CATS questions about experiences of being threatened, hit or hurt badly in the family, and questions about being pressured to forced to sexual things, were used as measures of physical abuse and sexual abuse exposure. Additional questions, based on the original ACE questionnaire were added to the CATS to measure emotional abuse, emotional neglect, physical neglect, domestic violence, parent substance abuse, and parental incarceration. Parental mental health problems and parental separation were measured elsewhere in the questionnaire. Exposure to each adversity (‘Yes’ or ‘No’) was counted across the 10 categories and responses grouped as ‘0 adversities’, ‘1 adversity’, ‘2 adversities’ and ‘3 or more adversities’.

Screening Measures for Autism Spectrum Disorder, Eating Disorders and Psychotic Like Symptoms

: The Modified Checklist for Autism in Toddlers, Revised (M-CHAT-R; Robins et al., 2014) and Autism Quotient (AQ-10) were used to identify children and young people at risk of autism spectrum disorder (ADS). The M-CHAT-R is a two-stage validated screening tool for toddlers aged between 16 and 30 months of age designed to identify children who may benefit from a more thorough developmental and autism evaluation. Previous research has demonstrated that participants with total scores ≥3 initially, and ≥2 after follow-up, were associated with a 47.5% risk of being diagnosed with ASD and a 94.6% risk of any developmental delay or concern (Robins et al., 2014). The NIYWS used the first stage screening tool to identify ASD characteristics.

The AQ is a parent report measure which can be completed about a child aged 4–11 years (AQ-10, child), or adolescent aged 12–15 years (AQ-10, adolescent), with suspected autism who does not have a learning disability (Allison et al., 2012). It includes 10 items covering five domains and has strong internal consistency (>.85). A cut-point of six on the AQ-10 has been shown to yield a sensitivity of .93, a specificity of 0.95, and a positive predictive value of 0.86, whilst a cut-point of six on the AQ-10 child, has a sensitivity of .95, a specificity of .97 and a positive predictive value of .94 (Allison et al., 2012).

Risk of eating disorders: The SCOFF questionnaire (Morgan et al., 1999) is a five-item screening scale which was used to assess the core features of anorexia nervosa (AN) and bulimia nervosa (BN) among 11–19 year olds. SCOFF is an acronym of key words in the five questions (Sick, Control, One, Fat, Food) but this was one of the language issues that raised some concern. Items are binary scored (1 present; 0 absent) and score of two or more is indicative of caseness. A recent meta-analysis of 25 validation studies (Kutz et al., 2020) identified a pooled sensitivity of .86 and specificity of .83. Kutz et al. (2020) concluded that the SCOFF is a highly sensitive screening measure for young women at risk for AN and BN but not necessarily for other eating disorders or groups.

Risk of psychotic like experiences: The Prodromal Questionnaire (PQ-16; Ising et al., 2012) was used to screen for unusual, or ‘psychotic-like’ experiences (PLEs) associated with the psychosis prodrome among 11–19 year olds. The PQ-16 is a self-report 16-item questionnaire consisting of a perceptual abnormalities or hallucinations subscale (9 items), and unusual thought content, delusional ideas or paranoia subscale (5 items), and two items related to negative symptoms. Initially the presence of unusual experiences is assessed using a binary response (1 True, 0 False), and then any items that are endorsed are rated in terms of the distress caused using a 4-point scale (0 No distress, 1 Mild distress, 2 Moderate distress, 3 Severe distress). In initial analysis the NIYWS applied the cut-off of ≥6 on the symptom score to identify young people at risk of PLEs.

Self-injury and suicidal thoughts or attempts: Self-injury and suicidal thoughts or attempts were assessed using selected questions from the Deliberate Self Harm Inventory (DSHI; Gratz, 2001) and the Suicide Behaviours Questionnaire-Revised (SBQR; Osman et al., 2001). The items are (1) ‘Have you ever intentionally (i.e. on purpose) cut your wrist, arms, or other area(s) of your body (without intending to kill yourself)? (or burned yourself with a cigarette, lighter or match; carved words, pictures, designs or other marks into your skin’ and (2) ‘Have you ever thought about or attempted to kill yourself’? Participants screened positive for self-injury and suicidal thoughts or attempts by answering ‘Yes’ to both questions.

Parent Mental Health, Exposure to Childhood and ‘Troubles’ Related Adversity Measures.

Parent mental health: The General Health Questionnaire (GHQ-12; Goldberg & Williams, 1988) was used to assess current mental health functioning. The GHQ-12 is a widely used screening measure for identifying possible non-psychotic mental health problems in the general. It is a 12-item self-completion questionnaire which yields a maximum score of 12 based on ‘GHQ scoring’ (0-0-1-1), with a score of four or more used to identify individuals with potential mental health problems. Parents were also asked about any past or current mental problems they had experienced and what, if any, diagnosis they had received.

Parent’s exposure to childhood adversity: Parents were asked questions about their exposure to 10 childhood adversities. Questions were based on the questions used in the original ACE study (Felitti et al., 1998), with some amendments to adapt the language to the NI context to shorten the question format. Exposure to each adversity (‘Yes’ or ‘No’) was counted across the 10 categories and responses grouped as ‘0 adversities’, ‘1 adversity’, ‘2 adversities’, ‘3 adversities’ and ‘4 or more adversities’.

Experiences of ‘The Troubles’ and paramilitaries: Two questions were asked relating to political violence in NI: (1) ‘How much are you aware of the Troubles in Northern Ireland’? (Not aware, a little bit, a moderate amount, Quite a bit, Extremely aware) and ‘Have the Troubles had any impact on your family’? (No impact, A little bit, A moderate amount, Quite a bit, An extreme impact). Four questions about paramilitary groups were also asked: (1) ‘Paramilitary groups create fear and intimidation in this area’/‘Paramilitary groups contribute to crime, drug-dealing and anti-social behaviour in this area’ (Strongly agree, Agree, Neither agree nor disagree, Disagree, Strongly disagree) and ‘Have you ever been threatened by paramilitaries in your area?/Have you ever been injured by paramilitaries in your area?’ (Yes, No).

### Data collection

Fieldwork took place between 1^st^ June 2019 and 19^th^ March 2020 and was conducted by a social research company, Perceptive Insight. All sampled addresses received an advance letter introducing the study which contained information on the background and purpose of the Youth Wellbeing Survey NI, as well as details of how the data would be collected. A telephone number and online link were provided to allow households with no eligible children or young people to inform the project team. A postcard was also included emphasising the importance of making contact if the household was ineligible. Respondents were asked to read an information sheet which outlined how their data would be handled, including how it was collected, analysed and stored.

Experienced interview staff received comprehensive training prior to fieldwork starting, which included briefings from the research team: additional support was available to interviewers during data collection should any concerns or queries arise. Interviewers were equipped with various information materials to hand out to participants, including parent and young person specific versions of the study information sheet. Interviewers were instructed to make a minimum of five calls to each address, with calls to be made at different times of the day and different days of the week (excluding Sundays).

For households with more than one eligible child or young person aged 2–19 years, the child or young person whose next birthday was closest was selected to take part in the study. If that child or young person declined to be interviewed, interviewers were not permitted to substitute this child or young person with another child from that household. Data were collected using computer-assisted personal interviewing (CAPI) with the majority of information being collected via self-completion. Parents were asked not to sit beside the child or young person as they were completing the survey and vice versa, so only the participant themselves knew how they were answering the questions. The average interview time was 34 minutes. As an incentive to encourage participation, and to acknowledge the time involved, the main respondent to the survey was given a £10 shopping voucher at the end of the interview.

### Ethical considerations

Given that a number of the survey questions had the potential to identify young people at risk, it was essential to strike a balance between offering a safe environment for participants to answer questions honestly, and without fear of repercussion, and taking action to safeguard participants where significant risks were identified. A clear and transparent protocol was developed to outline the consent process, participant anonymity and confidentiality (and its limitations) and the safeguarding procedures. Each member of the interview team was trained in the safeguarding protocol and each survey participant was provided with a list of helpline numbers for organisations providing information about mental health and crisis support. The helpline information also encouraged participants to contact their GP if they needed help and advice.

Careful consideration was given to the design of the survey and the answers to particularly sensitive questions were entered directly into a computer tablet by the participant so that their responses remained confidential. The data were then ‘locked’ to prevent the interviewer accessing the information. However, if a parent or child spoke directly to the interviewer and disclosed something that caused significant concerns about their safety (or someone else’s), the interviewers were advised to consider whether additional support (e.g. from mental health services or social services), beyond the signposting offered in the service information leaflet, was necessary. Perceptive Insight’s Safeguarding Lead was available to interviewers at all times during fieldwork, and the research teams’ clinical lead and Principal Investigator were both available to the fieldwork team when specific concerns were raised (this happened on only one occasion). Ethical approval was granted by the School of Social Sciences, Education and Social Work Research Ethics Committee, Queen’s University Belfast in June 2019.

## Results

The end of the fieldwork coincided with the onset of the COVID-19 global pandemic and ensuing UK national lockdown, at which point 21,730 main sample addresses had been issued and 3074 interviews completed. As the final sample closely matched the NI population in terms of geographical location and deprivation sample (Table 2), the decision was taken to end the fieldwork. Of the 21,730 addresses issued over the survey period, 79% were ineligible, primarily because there was no child or young person resident in the household (83%). A further 16% of addresses were deemed ineligible either because they could not be found, were vacant or non-residential, or because their status could not be confirmed during fieldwork period, despite repeated call-backs. Of the remaining 4621 eligible address, 1492 (32%) were refusals and 55 (1%) were instances where the selected respondent (either parent or young person) was unavailable during the fieldwork period. In total 3074 surveys were completed giving a response rate of 67%.

**Table 2.** Sample demographics and comparisons with NI population.

	% Of sample	% Of NI children Population	Difference in %
Gender^a^			
Male	51.4	51.8	0.4
Female	48.6	48.2	−0.4
Age^a^			
2	6.5	5.5	−1.0
3	7.7	5.7	−2.0
4	6.6	5.7	−0.9
5	5.9	5.7	−0.2
6	6.8	5.8	−1.0
7	5.9	6	0.1
8	5.9	5.9	0.0
9	6.6	5.9	−0.7
10	5.9	5.9	0.0
11	4.5	6	1.5
12	4.3	5.7	1.4
13	4.5	5.4	0.9
14	4.1	5.3	1.2
15	4.4	5.2	0.8
16	4.7	5.1	0.4
17	6.1	5.1	−1.0
18	4.9	5.1	0.2
19	4.7	5.1	0.4
Ethnicity^b^			
White	95.0	97.5	2.5
Other	5.0	2.5	−2.5
Family type^c^			
Married couple mjtitotoy family with dependent children	60.6	62.7	2.7
Cohabiting couple family	10.4	7.2	−3.2
Lone	29.0	30.1	1.1
Deprivation^d^ decile			
1 most deprived	10	10	0
2	10	10	0
3	10	10	0
4	10	10	0
5	9	10	1
6	10	10	0
7	11	10	−1
8	10	10	0
9	10	10	0
10 least deprived	11	10	−1
County^e^			
Antrim	36	36	0
Armagh	9	9	0
Down	30	30	0
Fermanagh	3	4	1
Londonderry	13	14	1
Tyrone	9	8	−1

Note:

^a^Comparison based on 2019 mid-year population estimates for 2–19 years (individual age by sex).

^b^Comparison based on 2011 Census data for ethnicity

^c^Comparison based on 2019 Labour Force Survey (family type with/without dependent children).

^d^Comparison based on proportion of residential addresses within each deprivation decile contained within the pointer database, after data linkage, at the time of sample selection.

^e^Comparison based on proportion of residential addresses within each county contained within the pointer database at the time of sample selection.

Table 2 shows the breakdown of participants by demographic variables compared to the NI population statistics. Comparisons with NI population data showed close similarities between the sample distribution and the population distribution, with some minor variation within child age by individual year. In keeping with the stratification process, the proportion of the sample by county and deprivation decile was almost identical to the proportion of the residential addresses by county and deprivation decile at the time of sample selection from the Pointer database.

Table 3 presents the percentage of participants who scored ‘High’ on the SDQ’s total ‘Difficulties’ and separate subscales, stratified by age group and gender. The overall prevalence estimates for ‘High’ total difficulties scores was 11.0%: conduct problems (9.9%), emotional problems (11.9%), hyperactivity (14.7%), peer problems (3.4%) and prosocial behaviour (4.7%). There were significantly more males with high levels of conduct problems (χ^2^ (2) = 21.00, *p* < .001), hyperactivity (χ^2^ (2) = 66.97, *p* < .001), peer problems (χ^2^ (2) = 6.114, *p* < .001) and pro social problems (χ^2^ (2) = 45.52, *p* < .001). The 5–10 years and 16–19 years age groups were significantly associated with emotional problems (χ^2^ (6) = 100.70, *p* < .001) whilst younger age groups were significantly associated with conduct problems (2–4, 5–10 years: (χ^2^ (6) = 104.36, *p* < .001) and pro social problems (2–4, 5–10 years: (χ^2^ (6) = 21.76, *p* < .01). Hyperactivity was significantly associated with the middle age groups (5–10, 11–15 years: (χ^2^ (6) = 38.15 *p* < .001) and peer problems with the older age groups (11–15, 16–19 years: (χ^2^ (6) = 154.98 *p* < .001). Although. There was no overall difference between males and females in relation to emotional problems, there were considerable variations within age or gender categories with significantly higher rates of emotional problems in 5–10 year olds males compared to females (χ^2^ (2, *N* = 1130) = 8.06, *p* = .018), and 16–19 year old female compared to males (χ^2^ (2, *N* = 627) = 26.34, *p* < .001)

**Table 3.** Proportion of sample categorised ‘high’ strengths and difficulties questionnaire total and subscale scores by age and gender.

	% Total	% Males				% Females			
		2–4 years (*N*=322)	5–10 years (*N*=602)	11–15 years (*N*=349)	16–19 years (*N*=312)	2–4 years (*N*=313)	5–10 years (*N*=529)	11–15 years (*N*=319)	16–19 years (*N*=312)
Total difficulties	11.0	6.8	21.1	11.7	9.4	6.4	8.7	7.8	8.4
Emotional symptoms	11.9	3.1	19.3	6.9	6.7	4.5	15.3	11.3	19.7
Conduct problems	9.9	14.3	15.0	11.5	4.8	14.4	7.6	5.3	2.9
Hyperactivity and inattention	14.7	10.2	24.8	22.1	16.1	6.1	8.7	11.9	11.9
Peer problems	3.4	0.9	3.7	5.7	6.4	1.0	2.3	3.1	3.9
Prosocial behaviour	4.7	8.7	7.3	3.4	6.4	4.5	2.5	3.1	1.0

The Mental Health of Children and Young People (*MHCYP*: 2017) survey in England also used the SDQ to assess participants across the broader dimensions of emotional and behavioural problems. Table 4 compares the SDQ means scores from the MHCYP (2017) England with the findings from NIYWB for the 5–10 year old and 11–16 year old age groups. It shows that the mean total difficulties scores for NI 5–10 year olds is slightly higher than for England, with NI boys in particular scoring higher on the Emotional symptoms, Hyperactivity/Inattention and Peer Problems subscales. The mean total difficulties scores for NI 11–16 year olds was higher than for England for both boys and girls. Among the NI sample the girls scored higher than the boys on Emotional symptoms and Prosocial Behaviours, and the boys scored higher on the Conduct Problems, Hyperactivity/Inattention and Peer Problems subscales.

**Table 4.** Strengths and difficulties questionnaire means (95% CI) scores from the Northern Ireland youth wellbeing survey and mental health of children and young people by age and gender.

	Total sample		Boys		Girls	
*5-10 Years*	UK (2017) (*N* = 1428)	NI (2020) (*N* = 1134)	UK (2017) (*N* = 711)	NI (2020) *N* = (604)	UK (2017) (*N* = 717)	NI (2020) (530)
Total difficulties	8.16 (7.81–8.51)	8.67 (8.24–9.09)	8.92 (8.39–9.45)	9.94 (9.31–10.560	7.32 (6.85–7.79)	7.22 (6.68–7.76)
Emotional symptoms	1.99 (1.87–2.11)	2.14 (2.00–2.29)	1.95 (1.80–2.11)	2.32 (2.12–2.53)	2.03 (1.85–2.20)	1.94 (1.74–2.15)
Conduct problems	1.49 (1.40–1.59)	1.45 (1.35–1.55)	1.68 (1.53–1.82)	1.65 (1.50–1.79)	1.29 (1.16–1.43)	1.23 (1.10–1.36)
Hyperactivity and inattention	3.45 (3.29–3.62)	3.51 (3.34–3.68)	3.93 (3.69–4.18)	4.18 (3.93–4.43)	2.92 (2.71–3.14)	2.75 (2.54–2.97)
Peer problems	1.22 (1.13–1.32)	1.54 (1.43–1.65)	1.36 (1.21–1.50)	1.77 (1.60–1.94)	1.08 (0.96–1.19)	1.28 (1.13–1.43)
Prosocial behaviour	8.73 (8.62–8.84)	8.40 (8.28–8.52)	8.41 (8.23–8.58)	8.03 (7.85–8.21)	9.09 (8.97–9.22)	8.82 (8.67–8.96)
	Total sample		Boys		Girls	
11–16 years	UK (2017) (*N* = 4228)	NI (2020) (*N* = 810)	UK (2017) (*N* = 2135)	NI (2020) (*N* = 432)	UK (2017) (N = 2093)	NI (2020) (*N* = 378)
Total difficulties	7.92 (7.40–8.45)	10.14 (9.68–10.60)	8.26 (7.46–9.1)	10.29 (9.63–10.94)	7.59 (7.02–8.16)	9.92 (9.28–10.56)
Emotional symptoms	2.21 (2.05–2.37)	2.97 (2.80–3.13)	1.91 (1.68–2.1)	2.51 (2.30–2.72)	2.51 (2.31–2.72)	3.49 (3.23–3.74)
Conduct problems	1.25 (1.12–1.38)	1.61 (1.49–1.73)	1.37 (1.17–1.6)	1.82 (1.64–2.00)	1.13 (1.00–1.27)	1.38 (1.22–1.54)
Hyperactivity and inattention	2.78 (2.59–2.96)	3.82 (3.64–4.01)	3.20 (2.90–3.5)	4.15 (3.89–4.42)	2.37 (2.15–2.58)	3.43 (3.18–3.68)
Peer problems	1.68 (1.53–1.83)	1.72 (1.59–1.84)	1.78 (1.54–2.0)	1.78 (1.60–1.97)	1.58 (1.41–1.74)	1.61 (1.45–1.78)
Prosocial behaviour	8.64 (8.50–8.78)	8.09 (7.97–8.21)	8.45 (8.24–8.70)	7.71 9 (7.54–7.88)	8.82 (8.66–8.99)	8.54 (8.38–8.69)

## Discussion

The Northern Ireland Youth Wellbeing Survey is the first ever survey measuring the mental health of children and adolescents in Northern Ireland. The large, stratified sample (*N* = 3074), and high response rate (67%) create a sample that is representative of children across NI in terms of age and sex, as well geographical locations and levels of deprivation. These data will provide an important opportunity to understand the mental health difficulties experienced by young people using high quality data that will inform clinical practice and policy. Uniquely, the Youth Wellbeing Survey reports on the prevalence of combined posttraumatic stress disorder and complex posttraumatic stress disorder, as well the prevalence of psychotic like experiences, for the first time in a general population sample of children and young people. It is also the first UK survey to report on the prevalence of adverse childhood experiences (ACEs) in a youth population, enabling further exploration of how these experiences relate to the development of mental health problems among this group.

Initial findings from the Strengths and Difficulties Questionnaire indicate that 11% of NI youth aged 2–19 years are at risk of emotional and behavioural problems with approximately 1 in 8 children and young people in Northern Ireland experiencing emotional difficulties, 1 in 10 conduct problems and 1 in 7 problems with hyperactivity. In keeping with previous research, rates of behavioural problems were higher among males, as well as more common in the younger age groups (Hamblin, 2016; Kessler et al., 2007). Whilst girls are typically more likely than boys to have depressive disorders and anxiety disorders (Hamblin, 2016; Kessler et al., 2007), there was no overall difference between males and females in relation to emotional problems in the NI sample. However, there was considerable variation within age and gender categories with significantly higher rates of emotional problems among females aged 16–19 years compared to males (6.7% vs 19.7), as well as higher rates of emotional problems among males aged 5–10 years compared to females (19.3% vs 15.3%).

Direct comparison of mean SDQ scores between the NIYWBS and the MHCYP (2017) England samples also showed that NI 5–10 year olds had slightly higher total difficulties scores than their English counterparts, primarily due to substantially elevated scores for 5–10 year old boys who had higher scores in relation to emotional difficulties, hyperactivity and peer problems. Average total difficulties scores were slightly higher for NI 11–16 year olds compared to their English peers, although the magnitude of these differences were not large.

Although the data are unique and the data collection process of high quality, there remain several limitations. The approach of the research team was based on the premise that the different perspectives on mental health (mainly bio-medical, psychological and social) are all important and necessary. As such, the survey was designed to try to collect data which would enable as comprehensive and multi-factorial exploration of the mental health of children and young people as possible. Inevitably, even with this broad scope, not all issues could be included and even the relatively high number that were included could not be explored in substantial depth. This reflects some of the more practical and ethical considerations of the survey design, including what is a reasonable length of interview, especially for children.

As with any research design, there are also potential sources of bias. Although this survey achieved a relatively high response rate, there is still the possibility that the sample who did participate are not precisely representative of those who decided not to participate and of the wider population. The standardised measures used, although well tested, do also have their limitations. Nonetheless, the data collected and the analyses completed to date are extremely useful for further developing our understanding of the mental health of children and young people with initial analyses of the SDQ results pointing to differences in the nature and distribution of emotional and behavioural problems among NI youth compared to UK peers. The findings highlight elevated difficulty levels in relation to emotional and behavioural problems among 5–10 year old boys and emotional problems among 11–16 year old girls that warrant further investigation and consideration in the context of research, policy and practice.
